# Examining the safety of relaxed drug monitoring for methotrexate in response to the COVID-19 pandemic

**DOI:** 10.1093/rap/rkac100

**Published:** 2022-12-01

**Authors:** Abdulrahman Shadeed, Leila Kattach, Sharlene Sam, Kalveer Flora, Ziad Farah

**Affiliations:** University of Hertfordshire, Hatfield, Hertfordshire, AL10 9AB, UK; Department of Dermatology, Guy's and St Thomas' NHS Foundation Trust, Great Maze Pond, London, SE1 9RT, UK; Department of Rheumatology, Northwick Park Hospital, London North West University Healthcare NHS Trust, Harrow, UK; Department of Rheumatology, Northwick Park Hospital, London North West University Healthcare NHS Trust, Harrow, UK; Department of Rheumatology, Northwick Park Hospital, London North West University Healthcare NHS Trust, Harrow, UK; Department of Rheumatology, Northwick Park Hospital, London North West University Healthcare NHS Trust, Harrow, UK

**Keywords:** MTX, blood monitoring, rheumatology, DMARDs, RA, safe monitoring, pharmacology

## Abstract

**Objectives:**

This is a retrospective study that set out to assess the safety, feasibility and cost savings of temporary relaxed blood test monitoring for patients on MTX under the rheumatology service that was rolled out during the coronavirus pandemic.

**Methods:**

This is a single-centre study that reviewed the blood tests of all patients who received an MTX prescription from the trust between December 2019 and November 2020. After the application of inclusion and exclusion criteria, the blood testing intervals and findings were analysed and collated. The cost of the blood tests was obtained from the laboratory.

**Results:**

A total of 1194 patients were identified as having received an MTX prescription. After applying inclusion and exclusion criteria, 462 patients were included. Of these, 395 (85%) patients had a blood test within the standard 3-month schedule and 67 had blood tests within the relaxed blood monitoring schedule. Six patients had an abnormality identified on their blood tests, but no harm was caused by any of these abnormalities. The intervention resulted in a cost savings of at least £1187 from the blood test costs alone.

**Conclusion:**

MTX is a widely used steroid-sparing agent that requires regular blood test monitoring to reduce adverse outcomes for patients. During extraordinary circumstances such as a pandemic, relaxing the interval between monitoring blood tests in stable patients is a feasible intervention. A relaxed monitoring blood test interval for a set period is safe, achievable and cost effective.

Key messages

- Methotrexate is a steroid-sparing agent that requires regular blood monitoring to reduce adverse outcomes for patients.
- During extraordinary circumstances, relaxing the blood testing interval in stable patients is a feasible intervention.
- A relaxed monitoring blood test interval for a set time is safe, achievable and cost effective.

## Introduction

MTX is the first-line treatment for the management of several rheumatological diseases including RA [1, 2]. It is currently one of the most effective and widely prescribed therapies. Furthermore, it is regarded as the ‘anchor therapy’ among the DMARDs [1, 3, 4] and is an important chemotherapeutic drug also used to treat different types of cancers (in much higher doses) and several skin diseases, including psoriasis [5].

MTX’s precise mechanism of action for the treatment of RA is not fully understood and it is believed that it may inhibit pyrimidine and purine synthesis, resulting in anti-inflammatory and immunosuppressive effects [6]. There are several side effects associated with the use of MTX and toxicity can occur even with lower doses. The most common MTX-associated adverse effects are those related to the gastrointestinal system, including hepatotoxicity, vomiting, nausea, stomatitis and loss of appetite [7]. Therefore, regular and careful follow-up consultations are required with a trained clinician and, according to Bedoui *et al.* [1], monitoring blood work is essential in mitigating the systemic risks associated with MTX.

The National Institute for Health and Care Excellence (NICE) guidelines recommend monitoring full blood count (FBC) and renal and liver profiles every 14 days until the desired therapeutic dose has been reached and established for 6 weeks [8]. It is then recommended that monthly blood tests should be performed for 3 months and then once every 3 months thereafter. For those patients at higher risk of toxicity, more frequent blood monitoring may be required. Moreover, the NICE also recommends that MTX monitoring be initiated and continued by a specialist until the patient is stable on their prescribed dose, after which a shared care protocol with their general practitioner (GP) may be considered with mutual agreement. This schedule for MTX blood monitoring is also endorsed by the British Society for Rheumatology (BSR) [9].

During the coronavirus disease 2019 (COVID-19) pandemic in early 2020, the BSR introduced guidance on identifying patients in England at increased risk of contracting the COVID-19 due to immunosuppressive medications related to their underlying rheumatological diagnosis. This was based on a risk-stratifying guide that classified patients into three categories: low, moderate and high risk. The latter included the patients that should self-isolate and shield, meaning they needed to always stay at home and avoid face-to-face contact. It proved problematic for them to have the recommended blood work schedule for MTX monitoring. Considering this, the BSR recommended temporarily relaxing the standard blood test monitoring schedule for stable and well patients on DMARD treatment during the COVID-19 pandemic, which included MTX. The BSR recommended that during the pandemic, patients on DMARDs as a single agent for the treatment of their rheumatological disease could undergo blood monitoring every 6 months once they were established on therapy, having completed the required blood for the initiation period [10].

London North West University Healthcare Trust’s (LNWH) MTX monitoring policy was in keeping with NICE and BSR guidelines before the pandemic [8, 9]. However, this policy was temporarily amended in response to the new recommendations by the BSR [10]. This change aimed to bring about several benefits, including reducing the general footfall in the hospital and the potential risk associated with travel and hospital attendance at the height of the pandemic. Furthermore, the change is intended to reduce pressure on primary care and phlebotomy services, which would also reduce expenditures across the sectors. A memorandum was issued by the Trust’s Drugs and Therapeutics Committee detailing the specific criteria that MTX patients would have to meet to be eligible for relaxed blood monitoring. The criteria were stable patients, defined as those who have been on their current treatment for >1 year, and patients who had completed the initiation blood work close monitoring and had subsequently been kept at the same dose for >6 weeks.

For patients who met these criteria, blood tests were relaxed to every 6 months as opposed to every 3 months. This enabled eligible patients to continue to receive their regular 3 month supply of MTX provided blood test results were available for review, had been performed within the last 6 months and were within the acceptable range for the patient.

Following the implementation of the temporary extended blood monitoring policy, this retrospective audit was conducted to identify the risk and/or potential harm to patients following the implementation of the new schedule as well as to review the local cost implications of blood testing.

## Methodology

A request was made to the pharmacy department for a list of all patients in receipt of MTX therapy across all specialities within the Trust, which totalled 1194 patients and was provided to the auditor (junior medical physician) on an Excel spreadsheet (Microsoft, Redmond, WA, USA). All prescriptions for these patients were issued by the Trust pharmacy department and no prescriptions were issued from outside the Trust. The information requested was obtained from the records held on the Trust pharmacy database. The sample was large and therefore we felt that records from multiple Trusts were not required for this review. Patients receiving MTX therapy for non-rheumatological diseases were then excluded and a total of 820 rheumatology patients remained. The blood results and patient letters of the 820 patients were reviewed over a time frame of 12 months, from December 2019 to November 2020 inclusive, and blood work was analysed from the Trust’s database as well as regional GP blood results via ICE OpeNet. If patients’ results were outside the parameters of one or more of the pre-set measures (see Table 1), then this was deemed as causing potential harm. These parameters are published in the BSR guidelines for monitoring patients on DMARDs, which recommend that the analysis of blood work should not be limited to the review of absolute figures, but also blood work trends [10].

**Table 1.** Blood test thresholds used to identify potential harm

Blood test results
ALT and/or AST >100 U/l
Creatinine increase >30% over 12 months and/or calculated GFR <60 ml/min/1.73 m^2^
Mean cell volume >105 f/l
Neutrophils <1.6 × 10^9^/l
Unexplained eosinophilia >0.5 × 10^9^/l

The inclusion criteria were as follows: rheumatology patients in receipt of oral or subcutaneous MTX therapy prescribed and dispensed from the LNWH pharmacy; patients in receipt of MTX only as a monotherapy (patients on combination systemic therapies were excluded) and patients who had had blood work performed within the specified time frame of 12 months (December 2019–November 2020 inclusive).

The exclusion criteria were as follows: patients on a treatment other than MTX for a rheumatologic disease, patients on MTX in addition to another systemic therapy, rheumatology patients in receipt of MTX who were initiated on MTX by a different speciality for the treatment of a non-rheumatological disease and patients who had a blood test gap of ≤3 months (standard protocol).

Once inclusion criteria were adhered to and exclusion criteria were applied, a total of 67 of the 820 patients in the database were eligible and included in the study.

Lastly, the auditor contacted the hospital blood laboratory and requested a breakdown of costs for each of the required blood tests to determine the potential cost savings from the relaxed blood test monitoring schedule. As this was a retrospective audit, and the relaxed drug monitoring was agreed to by the local Drugs and Therapeutics Committee of the Trust in response to the COVID pandemic, advice was sought and no formal ethical approval was deemed necessary.

### Results

**Figure 1.**
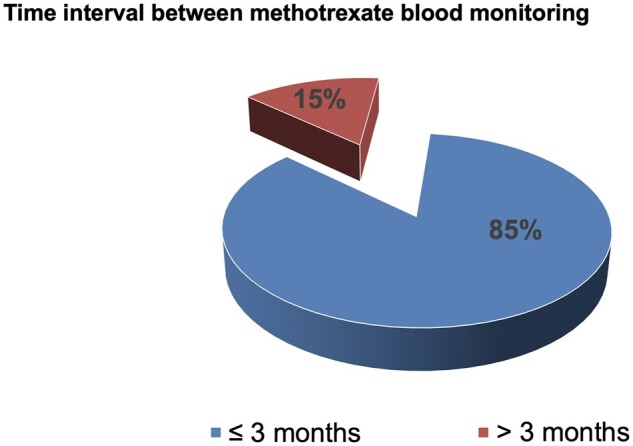
The proportion of patients who had a blood test within 3 months inclusive and >3 months

The study showed that 462 patients remained after application of the inclusion criteria. A total of 395 (85%) patients had a blood test up to 3 months inclusive. After the exclusion criteria had been applied, a total of 67 (15%) patients remained and had a gap in blood testing of >3 months (see Fig. 1). For those patients who met the criteria for the relaxed blood monitoring schedule, the mean duration between blood monitoring was 5.2 months and the median was 5 months. The minimum extended time frame was 4 months and the maximum extended time frame was 9 months, therefore the range for the time frame totalled 5 months.

## Results for patients who had a blood test at >3 months

**Figure 2.**
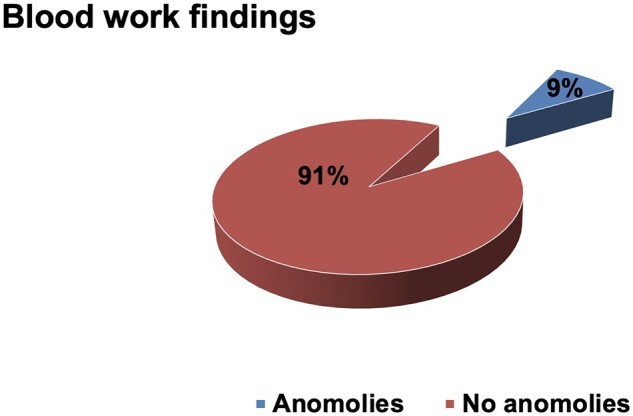
The findings from analysing the blood tests

The majority of patients [61 of 67 (91%) patients] had undergone relaxed blood test monitoring (see Fig. 2) and did not have any abnormalities in their blood work according to the thresholds used (see Table 1). A total of 6 (9%) patients had anomalies in their blood work that are detailed in Table 2. None of the six patients experienced actual harm.

**Table 2.** The identified blood work anomalies and their significance

Patient no.	Anomaly	Significance
1	ALT and/or AST >100 U/l	This result was not a significant elevation to justify further investigation and there was a previous high ALT in 2018 that was higher than this finding
2	Creatinine increase >30% over 12 months and/or calculated GFR <60 ml/min/1.73 m^2^	This was not a new finding and comparatively low values were seen on previous blood tests that did not justify further investigation
3	Mean cell volume >105 f/l	This result was not a new finding and was a chronic issue rather than acute and had been present for >10 years
4	Unexplained eosinophilia >0.5 × 10^9^/l	This was not a new finding and had been an ongoing anomaly since 2016
5	Unexplained eosinophilia >0.5 × 10^9^/l	This result was not a new finding and did not justify further investigation as per national haematology guidelines (British Society for Haematology, 2022)
6	Unexplained eosinophilia >0.5 × 10^9^/l	This was not a significant elevation to justify referral or a change to the management plan

The cost of individual blood tests carried out at LNWH are presented in Table 3. As a result of the extended blood monitoring, patients required fewer blood tests per annum. Every blood test costs £17.72 to process and this does not include other costs such as processing manpower and the clinician’s time, as well as the time and cost of travel for the patient. Therefore the minimum cost savings from the relaxed monitoring schedule was £1187 in blood test costs alone.

**Table 3.** Cost of blood tests

Profile	Test	Cost (£)
	FBC	3.41
Liver function tests	ALT	0.96
	Alkaline phosphatase	0.96
	Bilirubin	0.97
	Albumin	0.97
Urea and electrolytes	Urea	0.97
	Creatinine	0.95
	Potassium	0.96
	Sodium	0.96
Bone	Calcium plus adjusted calcium	0.98
	Albumin	
	ALP	
	Phosphate	0.97
	CRP	1.18
	ESR	3.48
Total cost		17.72

## Discussion

Initially, a large proportion of patients were eligible for the extended monitoring schedule and met the inclusion criteria. However, once exclusion criteria were applied only 15% met all inclusion and exclusion criteria. A total of 85% of patients were excluded for having additional blood tests during the relaxed blood monitoring period; the reason for this may have included the clinician’s continued adherence to pre-pandemic guidelines or additional blood work for other medical conditions requested by either a Trust clinician or their GP, as often the GP does not have access to blood test results taken in secondary care that have been ordered by secondary care. Moreover, GPs may not have been aware of the temporary relaxed monitoring schedule introduced by the BSR (2020) and continued adhering to standard good practice.

MTX is currently the most commonly prescribed DMARD for the treatment of rheumatic diseases [11–14] and has the most available safety data of all the DMARDs [9]. There is currently variation in physicians’ views regarding the monitoring requirements for MTX founded on personal experience in prescribing MTX. There are several published schedules for monitoring immune modulatory treatments across several specialities and countries that have been reviewed and modified over time [9]. Nevertheless, current practices are influenced by locally agreed guidance and national and international guidelines as well as pharmaceutical recommendations for MTX prescribing. These currently include, but are not limited to, the guidelines produced by the ACR, the EULAR, the BSR [9, 15–18], the British National Formulary (BNF) [19] and the Electronic Medicines Compendium (EMC) [20].

In the UK, previous BSR guidelines recommended drug-specific blood monitoring schedules that varied, and this was found to cause confusion among service users and clinicians in both primary and secondary care. Therefore the ‘guideline working group’ led by the BSR collectively endorsed a single recommendation for all DMARDs [9] and rheumatologists have used this document as a basis to determine local agreement and guidance.

## Liver monitoring

Guidelines for monitoring patients on MTX have an extensive history across different specialities and are founded on rare observations of serious adverse events. One of the main adverse effects of MTX is hepatotoxicity and slight elevations in aminotransferases are common.

After an extensive review of the available research on MTX, Ledingham *et al.* [9] concluded that patients who are at greater risk of liver toxicity from MTX will develop biochemistry abnormalities early on in treatment and treatment should be discontinued accordingly. Moreover, patients who remain on MTX and reach 12 months of therapy without abnormalities are, in effect, healthy MTX users and predictors of liver toxicity with patients on MTX include lack of folate supplementation and coexistent fatty liver disease. Concerning mitigating the risk of liver toxicity, the ACR recommends that patients receiving MTX treatment should have a liver enzyme blood test performed every 12 weeks after the initiation period [16]. Both the BNF [19] and EMC [20] recommend liver function tests every 2–3 months after initiation. The BSR recommends that the frequency of alanine transaminase (ALT) and/or aspartate transaminase (AST) and albumin testing after 3 months of a stable dose of MTX should be every 12 weeks, with more frequent monitoring in patients at higher risk of toxicity [9, 15]. In our study, only patients who were stable on their MTX treatment for >12 months were included in the relaxed monitoring schedule, and in none of these was any significant liver derangement or harm identified.

## FBC monitoring

MTX suppresses cell proliferation and thus routine haematologic monitoring is required [15]. The ACR recommends FBC tests every 8–12 weeks for months 3–6, then every 12 weeks thereafter [16]. Both the BNF [19] and EMC [20] recommend FBC testing every 2–3 months after initiation. The BSR recommends FBC tests be performed once the MTX dose is stable for 6 weeks, then monthly for 3 months and at least every 12 weeks thereafter [9, 15]. The most common FBC abnormality identified among the patients with relaxed blood test monitoring in our study was a slight increase in eosinophils, but no actual clinical harm was recorded. One patient had chronically elevated mean cell volume. In no patients was there any significant cytopaenia identified relating to relaxed blood test monitoring.

## Renal function monitoring

The BSR advises that creatinine/calculated glomerular filtration rate (GFR) testing be performed every 12 weeks [9]. The EMC [20] recommends renal function tests (including urinalysis) every 2–3 months and the BNF [19] recommends a renal function test every 2–3 months. Concerning MTX and its clearance by the kidney, if renal abnormalities are present, more frequent monitoring is suggested. Only one patient in our study had a significant increase in creatinine identified, but this was a patient with fluctuating renal function. Therefore, relaxing the blood test monitoring for this patient was unlikely to have been associated with any increased risk of harm. It is possible to argue, however, that in patients with a history of fluctuating renal function and on a trajectory towards progressive renal disease, it may be a safer option to maintain them on the conventional 3 month blood testing schedule.

## Ethnicity

The diversity of the population being treated with MTX therapy and how different ethnicities metabolize DMARDs should be considered. This review studied a diverse London population and Zamber *et al*. [21] found that Japanese patients may have distinct adverse event profiles, including disparities in routes of metabolism for DMARDs such as MTX. Ranganathan and McLeod [22] noted that there were differences between Caucasian, Black and Asian people and the way they metabolize MTX, although the studies reviewed were small. Helliwell and Ibrahim [23] highlighted a clear difference between ethnic groups concerning the tolerance of DMARDs and that genetic differences may be one of the contributing factors explaining the observed contrast.

## Current and future good practice

The current BSR guidelines by Ledingham *et al*. [9] suggest that FBC, creatinine/calculated GFR, ALT and/or AST and albumin should be performed every 12 weeks at a minimum and that tailored monitoring may be required for patients at greater risk of drug toxicity. Such risk factors include but are not limited to elderly patients, those with comorbidity and polypharmacy, patients with a history of drug-related toxicity [9] and patients for which there is concern about adherence.

The BSR suggests that stable patients can be considered for reduced frequency of blood monitoring and need to be assessed on a case-by-case basis; however, recommendations for extended time intervals with the exception of the pandemic have not been suggested [9]. Patient groups are in support of regularly reviewing monitoring frequency and research continues regarding MTX safety and blood monitoring. The current BSR guidelines [9] recommend nine monitoring blood tests in the first 12 months *vs* 14 in the previous guideline, and previous UK guidance recommended monthly monitoring in stable patients, in contrast to every 12 weeks in the ACR [17], demonstrating how research has influenced practice, as the BSR now advises 12 week monitoring [9]. The COVID-19 pandemic provoked rapid change to standard practice in order to keep the population safe.

Relaxed monitoring brings about many advantages, including but not limited to reduced patient visits to the hospital or to their GP practice, reduced pressure on phlebotomy services and also reduced healthcare costs across sectors. The pandemic altered the way the National Health Service delivers its care and some changes are likely to remain. Our study shows that extending blood monitoring did not cause harm to any of the patients who met the inclusion criteria and that during extraordinary circumstances like the pandemic, relaxing blood monitoring can be done safely and effectively. It also demonstrates that relaxed blood monitoring could in the future be considered a part of standard practice for monitoring stable patients on MTX in the absence of risk factors.

## Limitations

There were some limitations to the data collected. The guideline from the BSR [10] included recommendations for blood monitoring for rheumatology patients on DMARDs and this review only examined patients on MTX as a single agent. Moreover, no patients initiated on MTX under a speciality other than rheumatology were included despite patients also having a diagnosis of a rheumatology condition for which MTX may have been considered their rheumatological treatment. Furthermore, patients who had undergone any additional blood work for any other medical reason, including via their GP, were excluded, regardless of whether the blood tests were within their normal limits.

## Conclusion

This was a cross-sectional retrospective study to examine the safety of patients on MTX treatment following a relaxed blood monitoring schedule during the COVID-19 pandemic. The outcomes showed that no harm was observed in any of the patients included in this study. A multicentre, international and across-speciality retrospective study is required that includes all patients receiving MTX treatment to examine blood monitoring abnormalities, at what stage in the treatment they occurred and if any harm occurred. Furthermore, a prospective study observing stable and well rheumatology patients on MTX treatment as a single agent and following a standard (12 week) *vs* extended (24 week) monitoring schedule may support changes to BSR monitoring recommendations in the future.

While standard practice should remain, this study demonstrates that during extraordinary circumstances such as a global pandemic, the option to relax blood test monitoring is feasible, safe and cost effective.

## Data Availability

The datasets used during the current study are available from the corresponding author upon reasonable request.
